# Comparative Cytogenetic Study of Y Chromosomes in *Bovidae*: Insights from Morphological Analysis of European Bison, American Bison, and Domestic Cattle

**DOI:** 10.3390/ani15233442

**Published:** 2025-11-28

**Authors:** Marta Kloch, Magdalena Perlińska-Teresiak, Marlena Wojciechowska, Justyna Jazowska, Wanda Olech

**Affiliations:** 1 Department of Animal Genetics and Conservation, Warsaw University of Life Sciences-SGGW, Ciszewskiego 8, 02-786 Warsaw, Poland; 2 Faculty of Chemistry, Warsaw University of Technology, Noakowskiego 3, 00-664 Warsaw, Poland

**Keywords:** European bison, American bison, domestic cattle, cytogenetics, chromosome Y, *Bovidae*, *Bos*, *Bison*

## Abstract

In this study, we examined Y chromosome morphology in three species: European bison (*Bison bonasus*), American bison (*Bison bison*), and domestic cattle (*Bos taurus*). Peripheral blood lymphocytes were cultured, and C-banded metaphase preparations were analyzed to measure key Y chromosome parameters—absolute and relative length, absolute and relative area, and heterochromatin (C-band) content—using ImageJ software (ver. 1.54p). All traits deviated from normality (Shapiro–Wilk, *p* < 0.05). Non-parametric analyses revealed significant interspecific differences, with the strongest effects observed for C-band, followed by absolute and relative length. Principal Component Analysis (PCA) indicated a two-class structure, where domestic cattle formed a distinct cluster, while European and American bison grouped closely but remained separable. Linear Discriminant Analysis (LDA) achieved high classification accuracy (0.94), with misclassifications limited to American versus European bison. Generalized Linear Model (GLM) analyses further confirmed a strong species effect on C-band content (R^2^ = 0.916). These results identify the C-band as the most powerful discriminant, providing robust morphological differentiation among the three species and demonstrating the value of Y chromosome morphometry for comparative cytogenetic studies. Due to the limited sample size of American bison (n = 2), interpretations for this species should be made with caution.

## Simple Summary

Comparative analyses of the Y chromosome provide valuable insights into genetic diversity, species differentiation, and evolutionary history in *Bovidae*. In this study, we examined the Y chromosomes of European bison (*Bison bonasus*, n = 32), American bison (*Bison bison*, n = 2), and domestic cattle (*Bos taurus*, n = 6). Using classical cytogenetic methods (C-banding), we measured key structural features: length, relative length, area, relative area, and heterochromatin content. The analyses revealed consistent morphological differences among species, with the C-band identified as the most informative parameter. Multivariate statistical methods, including principal component and discriminant analyses, demonstrated that domestic cattle are distinct from both *Bison* species, while European and American bison are more similar yet still separable. The high classification accuracy confirmed the robustness of the applied markers. These findings highlight the potential of Y chromosome morphology as a powerful comparative approach for distinguishing closely related species and its relevance for biodiversity and conservation research.

## 1. Introduction

The *Bovidae* family comprises species of considerable ecological, cultural, and economic relevance. Within this family, the European bison (*Bison bonasus*), American bison (*Bison bison*), and domestic cattle (*Bos taurus*) represent a closely related group that diverged approximately one million years ago [1,2,3]. Their shared ancestry and well-documented history of hybridization have made them a valuable model for comparative research, including studies where genomic and cytogenetic data are incomplete. All three species share a conserved karyotype (2n = 60), and evolutionary analyses indicate substantial chromosomal similarity across *Bovidae* [4,5,6,7]. However, one notable exception is the Y chromosome, whose morphology differs strikingly between cattle and both *Bison* species: domestic cattle possess a submetacentric or metacentric Y chromosome [8,9,10], whereas the European and American bison consistently exhibit an acrocentric form [5,11,12].

These differences have prompted two major hypotheses regarding the origin of Y chromosome variability in *Bovidae*: structural rearrangements such as pericentric inversions or translocations, and evolutionary shifts in the abundance of repetitive DNA sequences [6]. A further hypothesis proposes that the acrocentric configuration represents the ancestral state within the *Bovinae* [5]. Because the Y chromosome is enriched in repetitive sequences and displays extensive heterochromatin, particularly in the pericentromeric region, it is highly sensitive to lineage-specific changes in repeat organization [13,14,15,16]. Such variability, even within closely related taxa, may provide informative cytogenetic markers for species differentiation and for studying male-specific evolutionary processes.

Despite advances in long-read sequencing, the Y chromosome remains one of the least resolved components of mammalian genomes. Several assemblies for domestic cattle have improved overall genome contiguity (e.g., ROSLIN_BTT_NDA1, ARS-UCD2.0), yet the repetitive structure of the Y chromosome has remained challenging to reconstruct [17]. Recently, Olagunju et al. [18] generated complete assemblies of the Y chromosomes of cattle and sheep, revealing extensive structural divergence and variable gene content. The Y chromosome of domestic cattle is 59.4 Mb long, contains roughly 50% repetitive sequences, includes 352 protein-coding genes and 50 pseudogenes, and its extensively expanded ~40 Mb ampliconic region (with numerous copies of *TSPY*, *HSFY*, *PRAME*, *ZNF280A/B*, and *RBMY*) is the primary feature shaping its structure and overall size [18]. Comparable assemblies for the European and American bison are currently unavailable; existing genomes are at the scaffold level and do not include a complete Y chromosome sequence [19,20,21]. Consequently, cytogenetic analyses continue to play an important role in characterizing Y chromosome organization in these species.

The European bison provides an additional context in which Y chromosome data may be particularly informative. The species experienced a severe demographic decline, with complete extinction in the wild by 1919, and subsequently recovered with only 12 individuals remaining [22,23,24]. This history has resulted in extreme genetic erosion, as documented at the molecular level [25,26,27,28,29,30,31,32] and likely reflected in Y chromosome variation as well. Understanding the structure and variability of the Y chromosome may therefore contribute to ongoing conservation and management efforts.

Studying Y chromosome morphology in *Bovidae* presents practical and methodological challenges. Access to material is often limited due to legal or conservation constraints, resulting in small sample sizes that reduce statistical power and make subtle intraspecific variation difficult to detect [33,34]. Moreover, morphometric analyses rely on precisely executed measurements that may introduce random variation related to landmark placement or scoring angle, even under standardized conditions [35,36,37]. Such residual variability does not systematically bias results but reinforces the need for rigorous analytical procedures, including nonparametric tests and cross-validated multivariate approaches [38], to ensure robust interpretation.

Against this background, the present study focuses on quantifying Y chromosome variation in the European bison, American bison, and domestic cattle. By integrating morphometric parameters, including Y chromosome length and area, expressed in both absolute and relative values, with heterochromatin content assessed through C-banding, the study aims to determine how these features contribute to species differentiation and what they reveal about Y chromosome evolution within the three studied *Bovidae* species. Multivariate analyses further assess the diagnostic value of these traits for distinguishing among the three taxa.

## 2. Materials and Methods

### 2.1. Research Samples

The material consisted of peripheral blood from European bison, American bison, and domestic cattle bulls collected in sterile, heparinized 9 mL tubes (Medlab, Raszyn, Poland). The material was collected between 2015 and 2022. The European bison samples were deposited in the European Bison Gene Bank at the Department of Genetics and Animal Conservation of the Warsaw University of Life Sciences, in accordance with the permit issued by the Regional Director for Environmental Protection in Warsaw for the keeping of parts of protected animal species dated 10 April 2014, ref. No. WPN-I.6401.90.2014.EB.1. Samples were collected during routine veterinary procedures, including microchipping, transport, release into the wild herd, or elimination for health reasons. The material from European bison came from 10 locations in Poland, breeding centers and wild herds (Białowieża n = 2; Bieszczady n = 8; Gołuchów n = 1; Muczne n = 8; Niepołomice n = 2; Pszczyna n = 6; Borecka Forest n = 2; Knyszyńska Forest n = 1; Smardzewice n = 1; Toruń n = 1). A total of 32 samples were collected from European bison. Based on their origin and pedigree data, it was determined that 16 samples came from males of the LB line, and 16 samples were collected from individuals from the LC line.

Research material from male American bison (n = 2) was obtained in Poland, where this species is listed as an Invasive Alien Species (IAS). Its legal status prohibits breeding and severely limits the availability of biological material. Blood samples were collected from the only existing captive group in the country during routine veterinary procedures. For comparison, material from domestic cattle bulls of the Limousine breed (n = 6) was collected at a slaughterhouse. Samples were obtained from domestic cattle bulls during routine slaughter at a licensed slaughterhouse. No animals were explicitly handled for this research, and no procedures beyond standard slaughter practices were performed. Therefore, no informed consent from animal owners or farms was required. All samples were transported to the laboratory under refrigerated conditions (4 °C).

### 2.2. Cell Culture

Cell culture was performed according to the standard cell culture protocol [39] with modifications: the incubation temperature was increased to 38.5 °C, and pokeweed lectin was used as a mitogen. Under standard culture conditions with phytohemagglutinin, the lymphocyte cultures exhibited very low mitotic activity, which informed our decision to modify the protocol by replacing phytohemagglutinin with pokeweed mitogen to enhance mitotic stimulation and chromosome quality (Supplementary Note S1). The lymphocyte cultures were supplemented with 8.5 mL of RPMI 1640 culture medium (Merck, Darmstadt, Germany), 10% fetal bovine serum (Merck, Darmstadt, Germany), pokeweed mitogen (100 µg/mL) (Merck, Darmstadt, Germany), and antibiotics (penicillin and streptomycin; 100 µg/mL) (Merck, Darmstadt, Germany). The blood was thoroughly mixed and then added to the culture to a final volume of 10 mL. The cultures were prepared in two replicates for each individual, labeled A and B. The tubes were incubated at 38.5 °C for 72 h in a horizontal position and gently mixed twice daily. One hour before the harvest, 1 μg/mL of colchicine (Merck, Darmstadt, Germany) was added to the tubes. Then, after one hour, the cultures were treated with a hypotonic solution—0.075 M KCl (Avantor Performance Materials, Gliwice, Poland) for 20 min at 38.5 °C in a water bath, and then fixed three times with freshly prepared, cold methanol/acetic acid (3:1). The fixed cell pellet was stored at −20 °C until the preparation of microscope slides.

### 2.3. Preparation of the Microscope Slides and C-Banding

Before preparing the microscope slides, the fixed, frozen cell pellets were centrifuged for 7 min at 1100 rpm, excess fixative was removed, and the remaining pellets were resuspended in freshly prepared and cold methanol/acetic acid (3:1). Cytogenetic preparations were made immediately before staining; the cell pellet was applied to SuperFrost microscope slides (Thermo Fisher Scientific, Waltham, MA, USA) previously stored at −20 °C and then air-dried. The C-banding method was used to identify constitutive heterochromatin in chromosomes. The preparations were subjected to a method described by Chaves et al. [40]. Briefly, slides were incubated with the restriction enzyme *Msp*I (Thermo Fisher Scientific, Waltham, MA, USA) at 37 °C for a minimum of 16 h. After this, the slides were rinsed in deionized water and air-dried. Next, the procedure for C-band staining, as described by Sumner [41], was applied with minor modifications. Specifically, the incubation time in HCl was shortened, and the duration in Ba(OH)_2_ was adjusted based on experimental optimization to prevent over-digestion and better preserve chromosome morphology. In brief, the preparations were incubated in a 0.2 M HCl (Avantor Performance Materials, Gliwice, Poland) solution for 30 min at room temperature, washed in deionized water (at room temperature), and incubated in a 5% Ba(OH)_2_ (Avantor Performance Materials, Gliwice, Poland) solution for 15 min at 50 °C (Supplementary Note S1). After this step, the preparations were rinsed in deionized water at 50 °C and incubated in a 2× saline-sodium citrate (Avantor Performance Materials, Gliwice, Poland) solution at 60 °C for 1 h. The preparations were then rinsed again in deionized water to remove any remaining salt sediment from the surface and air-dried. Finally, propidium iodide was used instead of Giemsa to enhance contrast and facilitate more precise visualization of chromosomes. Preparations were stained with propidium iodide (2 μg/mL) (Merck, Darmstadt, Germany) for 2 min, air-dried, and then mounted with VECTASHIELD^®^ Antifade Mounting Medium (Vector Laboratories, Newark, NJ, USA) to preserve the fluorescence.

### 2.4. Analysis of Microscopic Preparations and Y Chromosome Measurements

Metaphase spreads were observed and analyzed using a Leica DM3000 microscope (Leica Microsystems, Wetzlar, Germany), connected to a CV-M4+CL digital camera (JAI, Copenhagen, Denmark) and an EL6000 fluorescence illumination system (Leica Microsystems, Wetzlar, Germany). The prepared photographic documentation was analyzed using CytoVision Software, ver. 7.3.1 (Leica Microsystems, Wetzlar, Germany). In the initial stage, 20 metaphase plates from each male European bison (i.e., a total of 640 cells) were analyzed, 50 metaphase plates from each male American bison (i.e., a total of 100 cells), and 20 metaphase plates from each domestic cattle bull (i.e., a total of 120 cells), giving a total of 860 cells analyzed. The number of metaphase plates analyzed per species was determined based on preliminary analyses, which showed that these numbers provided stable mean values, allowing for a reliable morphometric characterization of the Y chromosome while balancing accuracy and experimental efficiency. Next, a representative group of cells was selected for each individual analyzed. Y chromosomes were identified based on their distinctive morphology and C-banding pattern, which allowed them to be clearly distinguished from autosomes in each species. Twenty Y chromosome measurements for each individual were converted into a dataset, from which measurements from four metaphase plates around the median representing the given set were selected. As a result, the following were analyzed: 8 American bison cells, 24 domestic cattle cells, and 128 European bison cells, of which 64 belonged to individuals from the LB line and 64 to individuals from the LC line (Table S1). The choice of 20 measurements per individual was supported by preliminary tests, which showed that additional metaphase analyses produced no meaningful improvement in the stability of mean values or variance estimates.

Y chromosome measurements were performed based on the chromosome length normalization system developed by Levan et al. [42]. Absolute chromosome length and area were measured; relative length and relative area were calculated as percentages of all chromosomes’ total length and area in the same metaphase plate. Based on the absolute area data, the area of constitutive heterochromatin within the Y chromosome was also measured. The measurements were performed using the IMAGEJ software [43] with the LEVAN plugin [44]. The analyses included, among others, adjusting the scale from pixels to µm or µm^2^ based on the scale from the photograph, determining the length of chromosomes by measuring the length of each of the two chromatids (function: straight line or segmented line) and averaging the results, as well as measuring the surface area of chromosomes and the surface area of the C-band using the threshold function.

### 2.5. Statistical Analysis

All statistical analyses were conducted in R (R Core Team, version 4.4.3) [45]. Data quality control included verifying naming consistency, detecting missing values and outliers, and checking for unit homogeneity. Distributional assumptions were tested with the Shapiro–Wilk test for normality and Levene’s test for homogeneity of variances (rstatix; ver. 0.7.2) [46]. As these assumptions were not met, interspecific comparisons were carried out using the Kruskal–Wallis test (rstatix), followed by Dunn’s post hoc tests with Benjamini–Hochberg correction for multiple comparisons (PMCMRplus (ver. 1.9.12) [47], rstatix). Effect sizes (η^2^(H)) were computed with the effect size package (ver. 1.0.1) [48].

Covariance structure was investigated using Principal Component Analysis (PCA [49], FactoMineR (ver. 2.12) [50], factoextra (ver. 1.0.7) [51]). Species classification was assessed by Linear Discriminant Analysis (LDA [52]; MASS [52]). Models were trained on two independent data partitions—an 80/20 train–test split and 10-fold cross-validation—to evaluate robustness and prevent overfitting. The optimal number of clusters was determined using the NbClust package (ver. 3.0.1) [53], which implements a comprehensive suite of indices and criteria, providing a robust, data-driven justification for cluster selection. Classification performance was evaluated using multiple complementary metrics: overall accuracy, balanced accuracy, sensitivity, specificity, and Cohen’s kappa [54,55].

Data were randomly partitioned into training (80%) and testing (20%) subsets using the initial_split() function from the rsample package (ver. 1.3.1), with stratification by species to preserve proportional representation across groups. A fixed random seed (set.seed = 123) ensured full reproducibility of the split. The stratified random division was methodologically justified despite the small overall sample size—particularly the limited number of American bison (*Bison bison*) specimens—because it prevents exclusion of the rarest class from either subset and allows each species to contribute to model training and evaluation.

For small and imbalanced datasets, stratified random sampling combined with repeated cross-validation is recognized as a best-practice approach [56]: it reduces sampling bias, maximizes data use efficiency, and provides a more reliable estimate of model generalization performance compared with deterministic or per-species partitioning. Therefore, ten-fold cross-validation was implemented as an additional safeguard against overfitting, ensuring that each observation was used for training and testing across iterations. This strategy enhances the stability of discriminant functions while maintaining the interpretability of species-level contrasts under constrained sample conditions.

Additionally, generalized linear models (GLM [57]; stats (ver. 4.4.3) [47], emmeans (ver. 1.11.2) [58]) were fitted to estimate the effect of species identity on each chromosomal trait. Model outputs included parameter estimates with standard errors, 95% confidence intervals, and Tukey-adjusted pairwise contrasts based on estimated marginal means.

All results were considered statistically significant at *p* < 0.05 after correction for multiple testing.

## 3. Results

### 3.1. C-Banding

**Figure 1:**
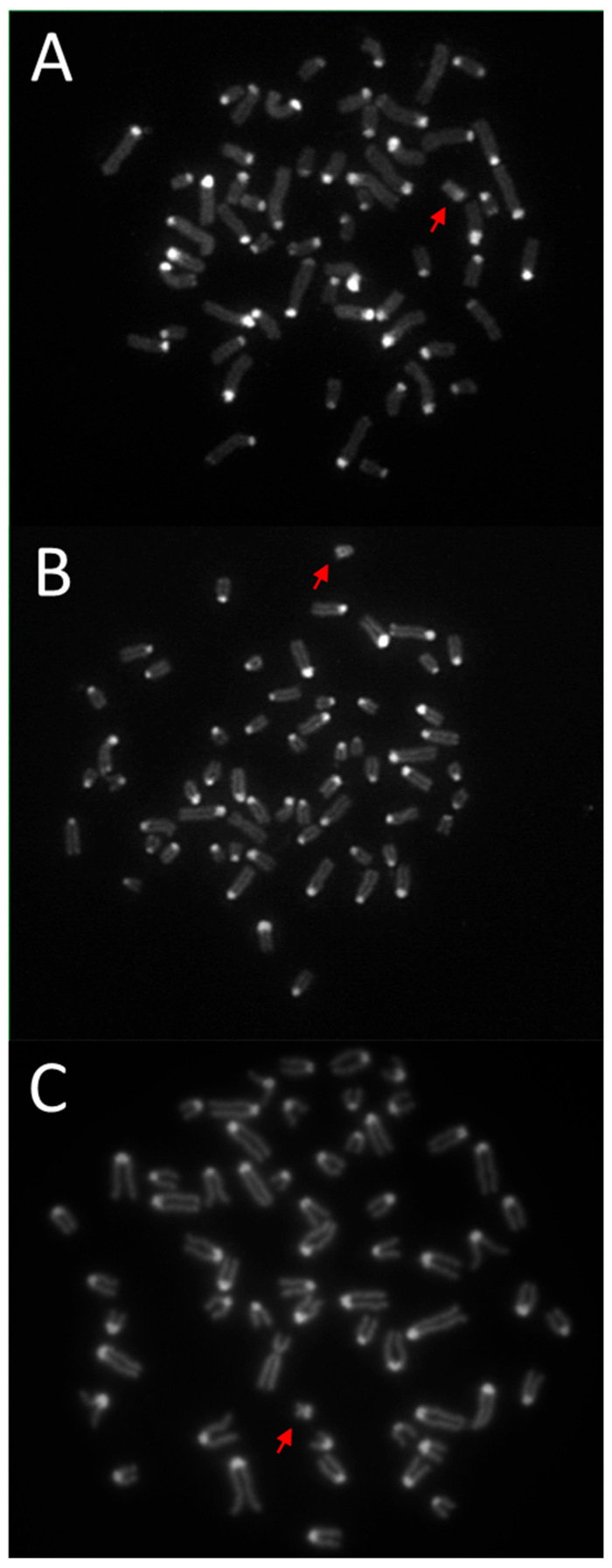
Results of the C-banding method with propidium iodide staining. Metaphase plates of: (**A**)—European bison (*Bison bonasus*), (**B**)—American bison (*Bison bison*), (**C**)—domestic cattle (*Bos taurus*). The Y chromosomes are marked with a red arrow. Magnification 100×.

The modified method for obtaining C-bands enabled the visualization of blocks of constitutive heterochromatin in the Y chromosomes of the studied species. The results of differential C-banding staining are presented in Figure 1. Based on the results obtained, it was determined that the C-band in the European and American bison is located at the end of the long arm of this chromosome (Figure 1A and Figure 1B, respectively). In contrast, in the Y chromosome of domestic cattle, a distinct C-band is visible in the short arm (Figure 1C). It is worth noting that the Y chromosome of the European and American bison differs in appearance from the other chromosomes. This chromosome is lighter in color, particularly evident when observing the original image, i.e., after fluorescent staining with propidium iodide. The negatives of the original photos are included in the Supplementary Materials (Supplementary Note S1).

### 3.2. Y Chromosome Measurements

The analyses demonstrated clear interspecific distinctions in the Y chromosome’s morphometric dimensions and C-banding patterns. Domestic cattle possessed visibly longer and more heterochromatin-rich Y chromosomes than the *Bison* species, reflecting their distinctive karyotypic organization. This was evident in the markedly higher C-band content in cattle (mean: 0.599 ± 0.049) compared to American bison (0.459 ± 0.037) and European bison (0.366 ± 0.017). In parallel, absolute chromosome length in cattle reached 2.057 ± 0.118 μm, distinctly exceeding values recorded in American (1.620 ± 0.147 μm) and European bison (1.560 ± 0.020 μm).

In contrast, the smaller Y chromosomes observed in American and European bison indicate a more compact chromosomal structure, with the European bison showing an exceptionally narrow range of values—for instance, the C-band IQR (interquartile range) spans only 0.352–0.378, highlighting remarkable chromosomal uniformity within the species.

Despite similar absolute surface areas across the three species (all around 1.74–1.78 μm^2^), relative measures revealed subtle yet consistent differences in chromosome proportion and heterochromatin content. The higher relative length of the American bison Y chromosome (mean 1.177 ± 0.064) may point to species-specific organization of centromeric or euchromatic regions. In contrast, the reduced C-band content in European bison (median: 0.362) suggests a less heterochromatinized and possibly more conserved Y-chromosome architecture. These descriptive outcomes are summarized in Table 1.

The unequal sample sizes among the studied taxa resulted from practical and legal constraints rather than sampling bias. The American bison is classified in Poland as an invasive alien species, severely limiting the collection and availability of biological material. Consequently, the number of American bison samples was considerably lower than that of domestic cattle and European bison. These discrepancies in group size, combined with non-normal data distributions and heteroskedasticity revealed by the Shapiro–Wilk and Levene’s tests, precluded the use of classical parametric procedures. To ensure reliable inference, non-parametric, robust, and cross-validated multivariate approaches were applied, emphasizing effect sizes and confidence intervals rather than sole reliance on *p*-values. While unequal *n* can reduce statistical power—particularly for the smallest group—it does not compromise the validity of the results when appropriate distribution-free methods and robust estimators are used.

**Figure 2:**
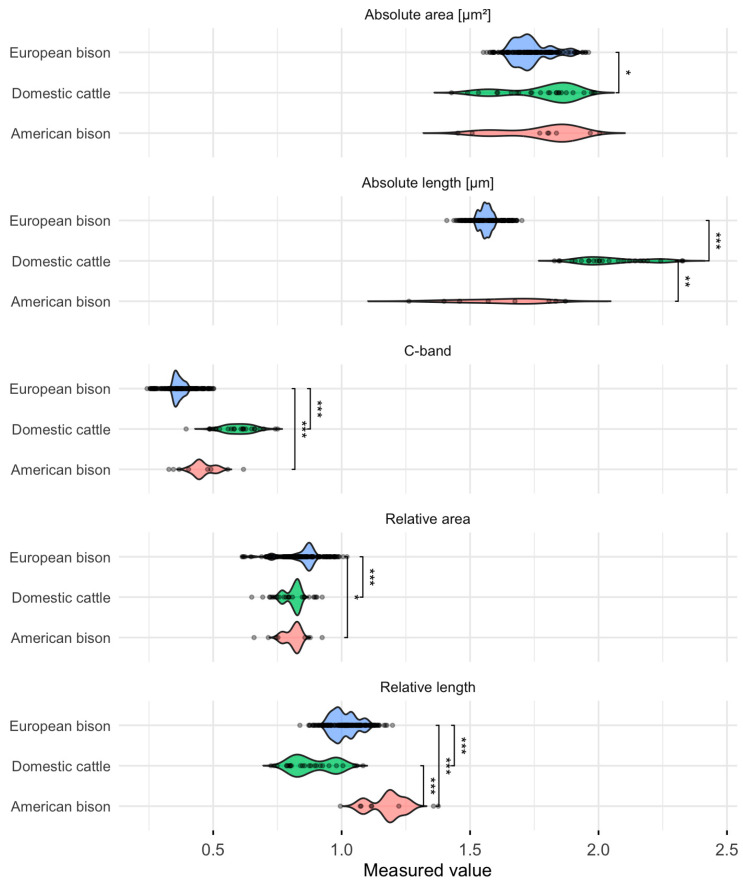
Violin plots illustrating variation in Y chromosome morphometric traits and C-band heterochromatin content in domestic cattle (*Bos taurus*), American bison (*Bison bison*), and European bison (*Bison bonasus*). Each panel represents the distribution and variability of a given trait within a species. Asterisks denote statistically significant pairwise differences according to Dunn’s post hoc tests with Benjamini–Hochberg correction * *p* < 0.05, ** *p* < 0.01, *** *p* < 0.001).

Nonparametric testing supported the descriptive observations. Kruskal–Wallis analyses revealed highly significant differences among species for all traits (*p* < 0.001). The assumption checks and global test statistics are summarized in Table 2, while the distribution of values with pairwise post hoc comparisons is visualized in Figure 2. Dunn’s tests with Benjamini–Hochberg correction confirmed that each species pair differed significantly, as indicated by the asterisks on the violin plots. Effect size estimates (η^2^(H)) further demonstrated that the observed differences were statistically significant and biologically meaningful, ranging from moderate to strong magnitudes.

**Figure 3:**
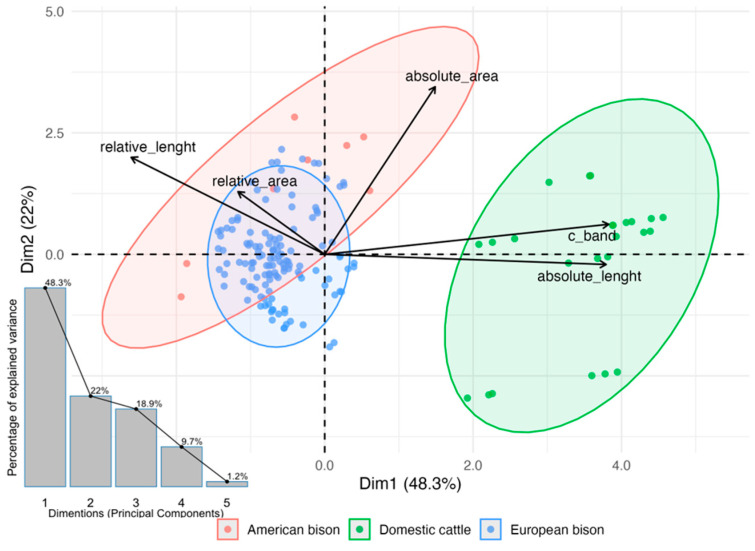
Principal component analysis (PCA) of Y chromosome morphometric traits and C-band heterochromatin content in domestic cattle (*Bos taurus*), American bison (*Bison bison*), and European bison (*Bison bonasus*). Black arrows represent variable loadings contributing to the first two principal components, which account for most of the total variance. Ellipses depict 95% confidence intervals around group centroids, showing clear separation among the three species. The inset scree plot illustrates the proportion of total variance explained by each principal component.

Principal Component Analysis (PCA) was employed to explore interspecific differentiation in Y chromosome morphometric and cytogenetic traits without assuming normality. The first two principal components explained most of the total variance and arranged the three taxa into well-separated clusters. Absolute and relative length contributed most strongly to PC1, whereas surface measures and C-band heterochromatin dominated PC2. In the PCA biplot (Figure 3), domestic cattle were characterized by larger absolute size and higher C-band content, American bison by the greatest relative length, and European bison by elevated relative surface values and reduced variability. Although PCA is mathematically unaffected by unequal sample sizes, visual separation can be influenced by the dominant contribution of larger groups; therefore, all variables were standardized to ensure comparable scaling across the three studied species.

**Figure 4:**
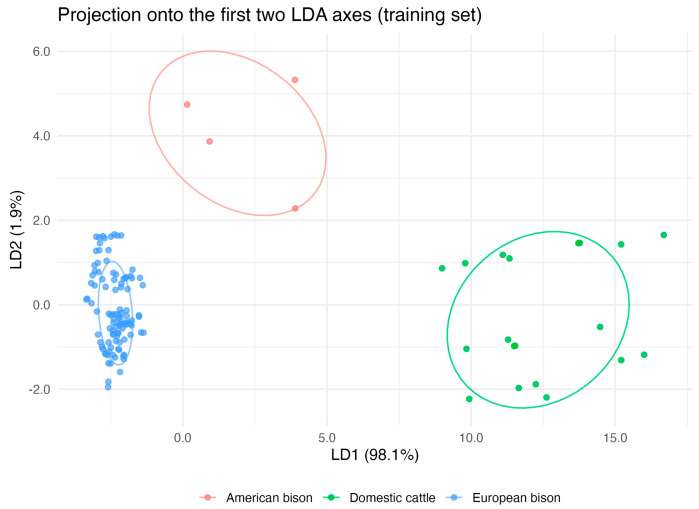
Linear discriminant analysis (LDA) of Y chromosome morphometric traits and C-band content in domestic cattle (*Bos taurus*), American bison (*Bison bison*), and European bison (*Bison bonasus*). The projection onto the first two discriminant functions reveals clear species separation, with 95% confidence ellipses around group centroids. The model demonstrated strong discriminatory performance, confirming reliable differentiation among species.

Linear Discriminant Analysis (LDA) confirmed these multivariate patterns and demonstrated strong discriminatory power of Y chromosome traits. The model achieved over 93% classification accuracy, and 10-fold cross-validation produced concordant results, confirming its stability. Sensitivity and specificity values were consistently high across species, indicating robust discrimination even for the small American bison sample (Figure 4). These findings reinforce the reliability of the interspecific differentiation suggested by PCA and validate the biological distinctness of the analyzed taxa despite unequal group sizes.

Generalized Linear Models (GLM) provided additional confirmation of these trends. In every model, species identity exerted a highly significant effect on the response variable (all *p* < 0.001). Estimated marginal means and post hoc contrasts mirrored the ranking patterns observed in the descriptive and multivariate analyses, further supporting consistent species-specific differentiation (Table 3). Robust variance estimation and model diagnostics were employed to account for heteroskedasticity and the unbalanced design, ensuring that the inference remained valid and biologically interpretable.

The combined use of non-parametric tests, PCA, LDA, and GLM ensured that statistical conclusions were resilient to deviations from normality and unequal sample sizes. This integrative and distribution-free framework allowed the detection of biologically meaningful patterns of Y chromosome variation even under the practical limitations imposed by the invasive status of the American bison.

## 4. Discussion

In the mid-19th century, unsustainable and uncontrolled hunting led to the near-total extermination of millions of American bison, causing a dramatic population collapse, particularly within the prairie subspecies. The survival of the American bison was ensured mainly through the efforts of five private breeders and the preservation of a free-ranging herd inhabiting the area now known as Yellowstone National Park [59]. Because private breeders maintained most surviving animals, frequent crossbreeding with domestic cattle occurred in an effort to enhance commercially valuable traits such as musculature, endurance, and resilience [59,60,61]. By 2007, only about 1.5% of American bison were estimated to be free from domestic cattle gene introgression [62]. A similar demographic crisis affected the European bison, which became extinct in the wild by 1919 due to hunting, habitat loss, and the effects of World War I [22]. At that time, only 54 individuals remained in captivity, and all modern European bison populations descend from just 12 founders [23,24]. This severe genetic bottleneck drastically reduced genetic diversity, a pattern also reflected in the variability of the Y chromosome. Currently, three Y chromosome haplotypes can be distinguished among males of this species, originating from the Plebejer (pedigree no. 45), Begründer (pedigree no. 15), and Kaukasus (pedigree no. 100) bulls. All occur in males of the LC line, whereas in males of the LB line only the Plebejer-derived Y chromosome is present. Their distribution was historically uneven (the Begründer haplotype was rare) [63], though planned breeding has partially altered this pattern.

Early cytogenetic studies of domestic cattle demonstrated that the short arm of the Y chromosome consists of material similar to centromeric regions of autosomes and contains highly condensed, repetitive DNA sequences [14,15,16,64]. The conservative nature of these sequences suggests an important functional role in maintaining proper Y chromosome structure and activity in *Bovidae* [65]. Substantial differences in Y chromosome morphology among cattle breeds have been documented, and Y chromosome polymorphism is heritable [66,67,68]. Reported estimates of longer Y chromosome length in domestic cattle range from 0.44–0.53% to 1.8–2.8% of the total genome length [69,70,71,72,73], whereas in the American bison, the relative length is approximately 1.9% [69], with absolute length reaching 1–2 µm [74]. Discrepancies between studies likely result from methodological variation and intrinsic Y chromosome polymorphism [58,71,72,75]. The observed diversity in Y chromosome structure among *Bos* and *Bison* species likely mirrors their evolutionary history and the dynamic processes shaping Y-linked sequences. Molecular studies indicate that the ancestral lineages of domestic cattle, European bison, and American bison diverged approximately one million years ago [1,2,3]. As in other mammals, the Y chromosome in these species comprises a recombining pseudoautosomal region and a male-specific region that does not recombine [76]. The absence of recombination in the male-specific region facilitates the accumulation of repetitive elements, gene duplications, and structural rearrangements, accelerating divergence between species and populations [77,78].

Although our data do not allow direct inference regarding specific chromosomal rearrangements, the observed differences in C-band content and Y chromosome length are consistent with previously proposed mechanisms involving structural and evolutionary modifications. In this study, this pattern positions the American bison as intermediate between domestic cattle and the European bison, reflecting their proximity within the *Bovidae* phylogeny. The differentiation in Y chromosome morphology and C-band distribution suggests that, despite shared evolutionary affinities, the two *Bison* species display distinct species-specific features, potentially reflecting shared ancestral variation rather than recent introgression from domestic cattle [59,62]. The acrocentric structure of the Y chromosomes in both European and American bison can complicate their identification using conventional staining methods. These chromosomes exhibit a distinctly brighter appearance under C-banding and propidium iodide staining, which highlights regions of constitutive heterochromatin, indicating a relatively high proportion of heterochromatin compared to other chromosomes in the metaphase plate. In contrast, the domestic cattle Y chromosome is typically submetacentric or metacentric, allowing for easier identification without the need for differential staining. Combining classical cytogenetic techniques with quantitative morphometry and multivariate statistics provides a strong methodological framework for resolving interspecific relationships and identifying potential hybridization events.

Differences in Y chromosome morphometry between domestic cattle and European bison reflect both intrinsic species characteristics and historical demographic processes. Domestic cattle exhibit longer Y chromosomes, whereas European bison show markedly reduced variability, consistent with their narrow genetic base. The interspecific variation in heterochromatin organization agrees with previous observations of C-band distribution in *Bos* and *Bison* [65], underscoring its potential as a diagnostic and evolutionary marker. Statistical analyses further confirmed pronounced interspecies differences across multiple traits. Both chromosome length and C-band organization proved particularly informative for distinguishing taxa, highlighting the value of Y chromosome morphometry as a diagnostic and phylogenetic tool in *Bovidae* research.

The main limitation of this study is the small sample size of the American bison, which is directly attributed to legal and logistical restrictions associated with collecting material from this invasive alien species. Although robust, non-parametric, and cross-validated statistical approaches were applied, the limited sample size inevitably reduces statistical power and may obscure subtle within-species variability. Consequently, interpretations concerning the American bison should be approached cautiously and verified as additional material becomes available. Similar challenges have been noted in population and molecular studies, where restricted sampling or environmental pressures can influence reproductive or genomic parameters [33,34].

Another potential source of uncertainty arises from the use of manual morphometric measurements. Despite rigorous calibration, repeated assessments, and standardized measurement criteria, minor inconsistencies in landmark placement or measurement angle can introduce random errors, especially when working with microscopic structures. Such manual variability is known to contribute predominantly to random rather than systematic bias in other biological measurement systems, thereby increasing residual variance without distorting true biological differences [35,36,37]. Any measurement noise in our dataset would therefore make the observed interspecific differences conservative rather than inflated. The use of digital image capture, repeated scoring, and statistical cross-validation further minimized these risks, following recommendations consistent with best practices for measurement reliability and data preprocessing [38].

Overall, our findings highlight the utility of Y chromosome morphology as a marker for interspecific differentiation within the studied species in the *Bovidae* family. Integrating cytogenetic, morphometric, and multivariate methods provides a robust framework for identifying diagnostic traits, assessing evolutionary relationships, and inferring limited introgression among closely related taxa. Future comparative studies incorporating Y chromosome morphometry with molecular methods, including PCR-based assays, sequencing, or fluorescence in situ hybridization, could offer additional resolution and further validate the diagnostic value of cytogenetic markers in *Bovidae*.

## 5. Conclusions

This study examined interspecific variation in Y chromosome morphology in domestic cattle, European bison, and American bison. The analysis showed consistent differences in Y chromosome length, heterochromatin content, and C-band content, indicating that these features provide reliable diagnostic value for distinguishing species. The small sample size of American bison, imposed by legal and logistical constraints, limits the statistical strength of the findings for this species and requires cautious interpretation. Despite this limitation, the results underscore the usefulness of Y chromosome morphology as a complementary approach in comparative cytogenetics and taxonomy, with potential relevance for evolutionary studies and conservation strategies.

## Figures and Tables

**Table 1:** Descriptive statistics (mean ± standard deviation (SD), median (IQR), and range) of Y chromosome morphometric parameters in American bison, domestic cattle, and European bison. Sample sizes are shown in column headings; “All” denotes pooled values across species.

	Domestic Cattle (n = 24)	American Bison (n = 8)	European Bison (n = 128)	All (n = 160)
C-band				
Mean ± SD	0.599 ± 0.049	0.459 ± 0.037	0.366 ± 0.017	0.406 ± 0.088
Median [IQR]	0.600 [0.564, 0.642]	0.448 [0.440, 0.477]	0.362 [0.352, 0.378]	0.370 [0.353, 0.397]
Min–Max	0.499–0.698	0.407–0.521	0.341–0.404	0.341–0.698
Absolute area [µm^2^]				
Mean ± SD	1.779 ± 0.131	1.779 ± 0.137	1.737 ± 0.067	1.745 ± 0.085
Median [IQR]	1.833 [1.726, 1.887]	1.833 [1.726, 1.887]	1.722 [1.684, 1.755]	1.726 [1.684, 1.814]
Min–Max	1.532–1.888	1.532–1.888	1.651–1.899	1.532–1.899
Absolute length [µm]				
Mean ± SD	2.057 ± 0.118	1.620 ± 0.147	1.560 ± 0.020	1.637 ± 0.186
Median [IQR]	2.020 [1.967, 2.121]	1.662 [1.524, 1.726]	1.561 [1.548, 1.575]	1.569 [1.550, 1.592]
Min–Max	1.931–2.250	1.364–1.785	1.525–1.593	1.364–2.250
Relative area				
Mean ± SD	0.810 ± 0.026	0.810 ± 0.028	0.836 ± 0.061	0.831 ± 0.057
Median [IQR]	0.821 [0.799, 0.832]	0.821 [0.799, 0.832]	0.868 [0.820, 0.876]	0.851 [0.807, 0.875]
Min–Max	0.762–0.835	0.762–0.835	0.651–0.903	0.651–0.903
Relative length				
Mean ± SD	0.888 ± 0.075	1.177 ± 0.064	1.009 ± 0.046	1.000 ± 0.079
Median [IQR]	0.862 [0.824, 0.970]	1.188 [1.154, 1.208]	0.991 [0.968, 1.039]	0.991 [0.966, 1.039]
Min–Max	0.802–0.992	1.075–1.259	0.948–1.098	0.802–1.259

**Table 2:** Results of Kruskal–Wallis tests assessing interspecific differences in Y chromosome morphometric and C-banding traits. All traits showed significant species effects (*p* < 0.001). For each variable, the table presents the effect size (η^2^(H)) with 95% confidence intervals, along with results of assumption checks (Shapiro–Wilk test for normality and Levene’s test for homogeneity of variances).

Variable	Kruskal–Wallis (*p*-Value)	Effect Size η^2^ (H)	Lower CI η^2^ (H)	Upper CI η^2^ (H)	Shapiro–Wilk (*p*-Value)	Levene’s Test (*p*-Value)
Absolute length [μm]	<0.001	0.384	0.270	0.520	<0.001	<0.001
Relative length	<0.001	0.310	0.190	0.430	<0.001	<0.001
Absolute area [μm^2^]	0.014	0.042	0.00	0.170	<0.001	<0.001
Relative area	<0.001	0.109	0.050	0.190	<0.001	0.108
C-band	<0.001	0.478	0.350	0.590	<0.001	<0.001

**Table 3:** Results of generalized linear models (GLM) assessing the effect of species identity on Y chromosome morphometric traits. The table presents regression coefficients with standard errors, *t*-values, significance levels, and pairwise post hoc contrasts (Tukey adjustment) with estimated marginal means (EMMeans) and 95% confidence intervals. All models indicated highly significant species effects (*p* < 0.001).

GLM Coefficient for C-Band					
Term	Estimate	Std. Error	T Value	95% CI	*p*-Value
(Intercept)	0.459	0.009	50.715	0.441–0.477	<0.001
Group Domestic cattle	0.140	0.010	13.428	0.120–0.161	<0.001
Group European bison	−0.093	0.009	−9.993	−0.112–−0.075	<0.001
Model: Gaussian identity (lm).					
**ANOVA (Type II) for the GLM**					
Term	DF	Sum Sq	F value	*p*-value	
Group	2	1.127	859.543	<0.001	
Residuals	157	0.103	NA	NA	
**Estimated marginal means (EMM) with Tukey group letters** Response: c-band; factor: group					
Group	Emmean		SE	95% CI	
Domestic cattle	0.599		0.005	0.589–0.610	
American bison	0.459		0.009	0.441–0.477	
European bison	0.366		0.002	0.361–0.370	

“NA” (“not available”).

## Data Availability

The data presented in this study are available on request from the corresponding author.

## Supplementary Materials

The following supporting information can be downloaded at: https://www.mdpi.com/article/10.3390/ani15233442/s1, Supplementary Note S1: Figure S1: Bison bonasus, Figure S2: Bison bison, Figure S3: Bos taurus; Table S1_Raw data.

## Abbreviations

The following abbreviations are used in this manuscript:

LB	European bison Lowland line
LC	European bison Lowland-Caucasian line
IAS	Invasive Alien Species
PCA	Principal component analysis
LDA	Linear discriminant analysis
GLM	Generalized linear models
IQR	Interquartile range
