# Supplementary material for: Comparative Cytogenetic Study of Y Chromosomes in Bovidae: Insights from Morphological Analysis of European Bison, American Bison, and Domestic Cattle
Source: Animals (Basel). 2025 Nov 28;15(23):3442. doi: 10.3390/ani15233442 (PMC12691336; doi:10.3390/ani15233442)
Supplement: Supplementary file 1 [file animals-15-03442-s001.zip › Supplementary Note S1.pdf]

2. Materials and Methods

2.2. Cell culture

Initially, cell culture was conducted according to a standard cell culture protocol at 37°C (Moorhead et al., 1960). Phytohemagglutinin was used as the growth factor (mitogen). Using this protocol, a very low mitotic rate was observed after the cell culture. The results were unsatisfactory, so a decision was made to modify the protocol. Two modifications were made: the incubation temperature was increased to 38.5°C (to approximate the body temperature of the animals from which the research material was collected), and an extract from pokeweed (American pokeweed) was used as the growth factor. This change in growth factor was also dictated by the fact that pokeweed stimulates T and B lymphocytes, whereas phytohemagglutinin stimulates only T lymphocytes (Lawce and Brown, 2017). In addition, lymphocyte cells usually grow in suspension, so to ensure even cell distribution and proliferation activation without disturbing the culture's integrity, gentle mixing was implemented (twice a day).

2.3. Preparation of the microscope slides and C-banding

The modifications were made to the Sumner (1982) protocol. These modifications aimed to optimize the quality of metaphase preparations under our experimental conditions. In particular, the incubation time in HCl was shortened, and the duration in $Ba(OH)_2$ was adjusted based on experimental optimization to prevent excessive digestion and better preserve chromosome morphology. Compared to Sumner's procedure, we shortened the incubation times to 30 minutes in HCl (instead of 1 hour) and 15 minutes in $Ba(OH)_2$ (within the original range of 5–15 minutes), while maintaining 1 hour of incubation in 2×SSC. The staining, however, was performed according to Chaves et al. (2000), using propidium iodide instead of Giemsa to increase contrast and facilitate more accurate visualization of chromosome structures. These adjustments were therefore driven by the need to adapt the protocol to our material and ensure high-quality cytogenetic analysis.

3. Results

3.1. C-banding

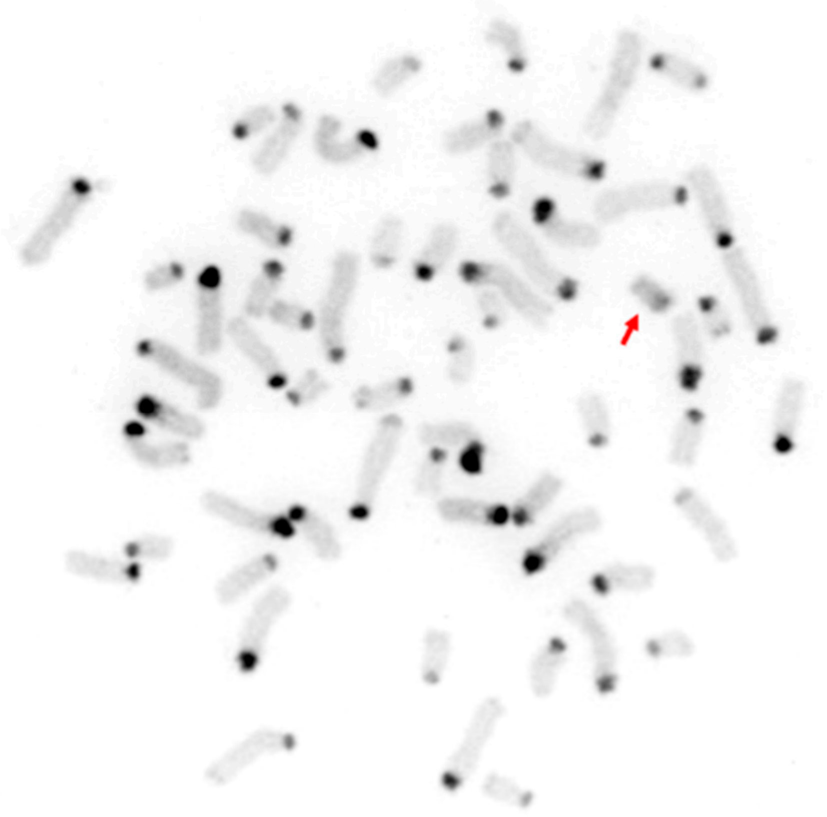

Figure S1. Results of the C-banding method with propidium iodide staining (the negative of the original photograph). Metaphase plates of European bison (*Bison bonasus*). The Y chromosome is marked with a red arrow. Magnification 100×.

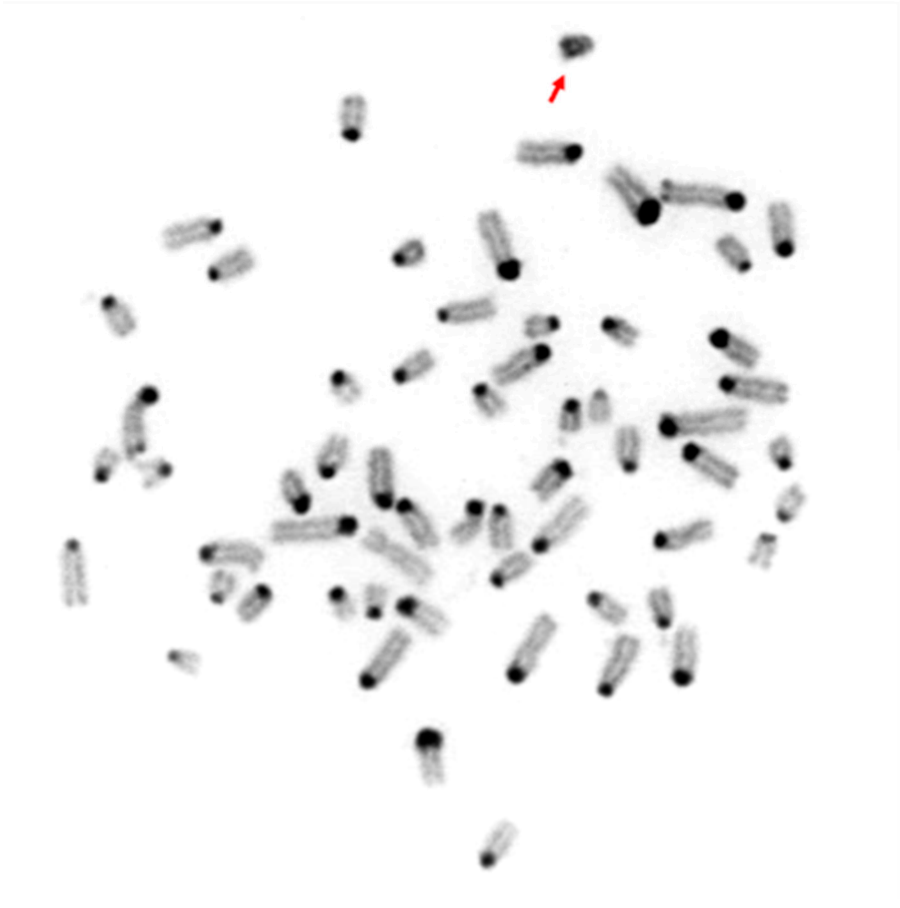

Figure S2. Results of the C-banding method with propidium iodide staining (the negative of the original photograph). Metaphase plates of American bison (*Bison bison*). The Y chromosome is marked with a red arrow. Magnification 100×.

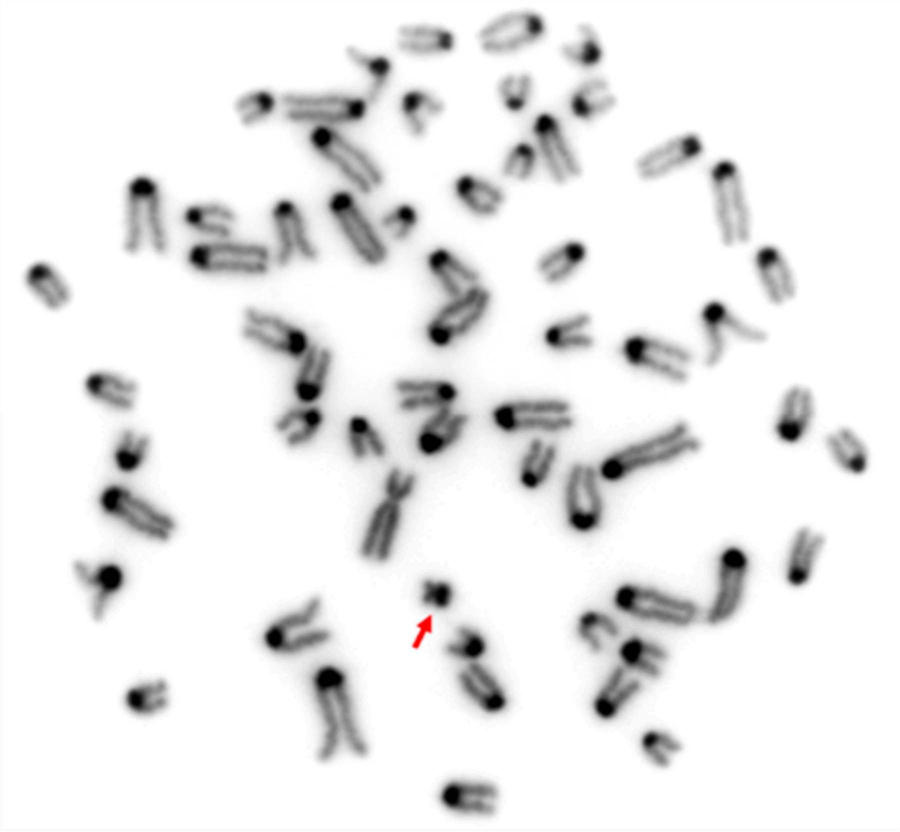

Figure S3. Results of the C-banding method with propidium iodide staining (the negative of the original photograph). Metaphase plates of domestic cattle (*Bos taurus*). The Y chromosome is marked with a red arrow. Magnification 100×.

References

Moorhead, P.S.; Nowell, P.C.; Mellman, W.J.; Battips, D.M.; Hungerford, D.A. Chromosome preparations of leukocytes cultured from human peripheral blood. *Exp. Cell Res.*, **1960**, *20*, 613-616. DOI: 10.1016/0014-4827(60)90138-5.

Lawce, H.J.; Brown, M.G. (2017). Peripheral blood cytogenetic methods. In: Arsham, M.S., Barch, M.J., Lawce, H.J. (editors). The AGT Cytogenetics Laboratory Manual. 4th ed. Hoboken, New Jersey, USA, pp. 213–300. DOI: 10.1002/9781119061199.

Sumner, A.T. A simple technique for demonstrating centromeric heterochromatin. *Exp. Cell Res.*, **1972**, *75(1)*, 304-305. DOI: 10.1016/0014-4827(72)90558-7.

Chaves, R.; Heslop-Harrison, J.S.; Guedes-Pinto, H. Centromeric heterochromatin in the cattle rob(1;29) translocation: α-satellite I sequences, in-situ MspI digestion

patterns, chromomycin staining and c-bands. *Chromosome Res.*, **2000**, *8(7)*, 621-626. DOI: 10.1023/A:1009290125305.
